# The Effect of Hyperglycaemia on *In Vitro* Cytokine Production and Macrophage Infection with *Mycobacterium tuberculosis*

**DOI:** 10.1371/journal.pone.0117941

**Published:** 2015-02-09

**Authors:** Ekta Lachmandas, Frank Vrieling, Louis G. Wilson, Simone A. Joosten, Mihai G. Netea, Tom H. Ottenhoff, Reinout van Crevel

**Affiliations:** 1 Department of Internal Medicine, Radboud University Medical Centre, Nijmegen, The Netherlands; 2 Radboud Centre for Infectious Diseases, Radboud University Medical Centre, Nijmegen, The Netherlands; 3 Department of Infectious Diseases, Leiden University Medical Centre, Leiden, The Netherlands; University of Maryland, UNITED STATES

## Abstract

Type 2 diabetes mellitus is an established risk factor for tuberculosis but the underlying mechanisms are largely unknown. We examined the effects of hyperglycaemia, a hallmark of diabetes, on the cytokine response to and macrophage infection with *Mycobacterium tuberculosis*. Increasing *in vitro* glucose concentrations from 5 to 25 mmol/L had marginal effects on cytokine production following stimulation of peripheral blood mononuclear cells (PBMCs) with *M. tuberculosis* lysate, LPS or *Candida albicans*, while 40 mmol/L glucose increased production of TNF-α, IL-1β, IL-6 and IL-10, but not of IFN-γ, IL-17A and IL-22. Macrophage differentiation under hyperglycaemic conditions of 25 mmol/L glucose was also associated with increased cytokine production upon stimulation with *M. tuberculosis* lysate and LPS but in infection experiments no differences in *M. tuberculosis* killing or outgrowth was observed. The phagocytic capacity of these hyperglycaemic macrophages also remained unaltered. The fact that only very high glucose concentrations were able to significantly influence cytokine production by macrophages suggests that hyperglycaemia alone cannot fully explain the increased susceptibility of diabetes mellitus patients to tuberculosis.

## Introduction

Type 2 diabetes mellitus (DM) has been increasingly recognized as an important risk factor for tuberculosis (TB). Epidemiological studies have demonstrated that adults with diabetes have a significantly increased risk of developing active TB [1] and it is estimated that globally 15% of TB cases are attributable to DM [2]. The global prevalence of DM will rise by an estimated 55% over the next 20 years, with the largest increases in TB endemic regions of Africa and Asia [3]. As a result, DM will become an increasingly important factor contributing to the sustained TB epidemic [4,5].

The causative pathogen of TB, *Mycobacterium tuberculosis* (MTB), primarily infects phagocytic cells of the lung, such as alveolar macrophages. The early stage of infection is characterised by the recruitment and accumulation of various innate immune cells at the site of infection including neutrophils, dendritic cells and interstitial macrophages, the latter of which subsequently become infected by the growing population of mycobacteria and ultimately develop into bacteria-sequestering granulomas [6,7]. Effective immunity against MTB is dependent on the production of pro-inflammatory cytokines like interferon-γ (IFN-γ) and tumour necrosis factor-α (TNF-α) [8–11], whilst anti-inflammatory cytokines such as interleukin-(IL)-10 can attenuate the anti-bacterial immune response [10,12].

It has been hypothesised that alterations in the immune response of patients with diabetes give rise to either an enhanced susceptibility to infection or accelerated progression towards active TB disease [5]. A possible explanation for these immunological changes is chronic hyperglycaemia, a hallmark of DM. Various studies have demonstrated that diabetes and hyperglycaemia in particular is associated with a compromised innate immune response which includes impairments in phagocytosis, cytokine secretion and macrophage activation [13–18]. Other studies that have investigated the adaptive arm of the immune response have yielded conflicting results when comparing differences in cytokine production between diabetes patients with or without TB [19–21]. These inconsistent data illustrate both the complexity of the interaction between TB and DM and reveal limitations in the comparability of studies that use divergent methods, such as differences in cellular origins and patient populations.

To better understand the effects of hyperglycaemia on the innate immune response during concurrent diabetes and tuberculosis, we investigated whether elevated concentrations of glucose could directly regulate the functional capacities of human macrophages *in vitro*. We initially determined the effects of hyperglycaemia on the cytokine response of PBMCs and macrophages after stimulation with bacterial lipopolysaccharide (LPS) or whole pathogen lysates, and later in alternatively activated (M2) macrophages upon infection with the MTB strain H37Rv. Finally, we assessed the phagocytic ability of hyperglycaemic M2 macrophages and studied their capacity to support mycobacterial outgrowth.

## Materials and Methods

### Ethics Statement

PBMCs were isolated from buffy coats donated after written informed consent by healthy volunteers to the Sanquin Bloodbank (http://www.sanquin.nl/en/) in Nijmegen and Leiden. Blood was collected anonymously which, according to institutional ethical policy, does not require a separate review by the Ethical Committee. Experiments were conducted according to the principles expressed in the Declaration of Helsinki.

### Healthy Volunteers

Since blood donations were anonymous no tuberculosis skin test or IFN-γ release assay could be performed but the incidence of TB in the indigenous Dutch population is extremely low (4/100,000) and Bacillus Calmette-Guérin (BCG) vaccination is not part of the routine vaccination program. The incidence of diabetes mellitus in the Dutch population for persons under 25 years of age is less than 1% and under 45 years of age is about 1.5%. Since the average age of blood donors in these experiments is approximately 45 years [22] we expect that almost none of them would have diabetes mellitus which would otherwise act as a confounding factor [23].

### Cytokine Stimulation Experiments

Isolation of peripheral blood mononuclear cells (PBMCs) was performed by differential centrifugation over Ficoll-Paque (GE Healthcare) within 0–2 hours of collection. After counting (Casy Counter) cells were adjusted to 5 × 10^6^ cells/mL were suspended in glucose free RPMI 1640 (Gibco) supplemented with 50 mg/mL gentamicin (Lonza) and 2 mM L-glutamine (Life Technologies). 100 μL of PBMCs was incubated in flat bottom 96-well plates (Greiner) with varying final glucose concentrations from 5 mmol/L to 40 mmol/L D-glucose (Sigma-Aldrich) in glucose-free RPMI, and stimulated with 1 μg/mL of H37Rv lysate, 1 × 10^6^ microorganisms/mL of heat-killed *Candida albicans* (ATCC MYA-3573 (UC 820)) or 10 ng/mL LPS (Sigma-Aldrich, E. coli serotype 055:B5). The plates were incubated for 24 h, 48 h or 7 days at 37°C in a 5% CO_2_ environment. Alternatively, 100 μL of PBMCs (5 × 10^6^/mL) was incubated for 1 h at 37°C in 5% CO_2_ and adherent monocytes were selected by washing out non-adherent cells with warm PBS. Adherent monocytes were differentiated into M2 macrophages (n = 18) in 10% human pooled serum and 50 ng/mL M-CSF (R&D Systems) or M0 macrophages (n = 23) in 10% human pooled serum for 6 days. Monocytes were differentiated in the presence of 5 mmol/L or 25 mmol/L glucose. In some cases monocytes were differentiated in 5 mmol/L glucose with 20 mmol/L mannitol (Sigma-Aldrich) to control for effects of osmolarity on cytokine production. Media containing M-CSF and/or serum and glucose were refreshed on day 3 of differentiation. Differentiated macrophages were stimulated on day 6 with RPMI (negative control), 1 μg/mL H37Rv lysate or 10 ng/mL LPS (positive control). Cell culture supernatants were collected after 24 h.

### Cytokine Measurements

Cell culture supernatants were collected and stored at -20°C for cytokine measurements, which were performed by ELISA: IL-1β, TNF-α, IL-17A, IL-22 (R&D Systems); IL-6, IFN-γ and IL-10 (Sanquin).

### H37Rv Lysates and Culture

Cultures of H37Rv MTB were grown to mid-log phase in Middlebrook 7H9 liquid medium (Difco, Becton-Dickinson) supplemented with oleic acid/albumin/dextrose/catalase (OADC) (BBL, Becton-Dickinson), washed three times in sterile saline, heat killed and then disrupted using a bead beater, after which the concentration was measured using a bicinchoninic acid (BCA) assay (Pierce, Thermo Scientific).

### H37Rv Infection of M2 macrophages

CD14^+^ monocytes were isolated from PBMCs by magnetic cell sorting with anti-CD14^-^coated beads (Miltenyi Biotec) and seeded in tissue culture-treated flasks (Corning). After 6 days of differentiation in the presence of 50 ng/mL M-CSF, M2 macrophages were harvested using trypsin and transferred to tissue culture-treated 24-well plates (Corning) with 300,000 macrophages per well. Macrophages were incubated O/N at 37°C in 5% CO2 and subsequently infected with the H37Rv strain of MTB. Mycobacterial cultures were diluted to pre-log phase density one day before infection to ensure that the bacteria were in the log phase of the growth curve. Bacterial density was determined by measuring optical density at 600 nm (OD-600) and the bacterial suspension was diluted to a concentration of 30 x 10^6^ bacteria/mL (MOI 10:1). 100 μL of the bacterial suspension was added to the cell cultures, after which the plates were centrifuged for 3 minutes at 800 rpm and incubated at 37°C in 5% CO_2_. After 60 minutes the plates were washed with culture medium containing 30 μg/mL gentamicin and subsequently incubated O/N at 37°C in 5% CO_2_ in medium containing 5 μg/mL gentamicin. Supernatants were collected and filtered before cytokine measurements. M2 macrophages were lysed in water for 5 minutes and plated on Middlebrook 7H10 agar (Difco, Becton-Dickinson) supplemented with OADC. CFUs were determined after 2–3 weeks.

### Cell viability assay

Macrophage viability during prolonged H37Rv infection was assessed by using a LDH cytotoxicity kit according to the manufacturer’s instructions (Pierce, Thermo Scientific). For each experimental condition 50 μL of freshly harvested supernatant was incubated with 50 μL LDH reaction mixture for 30 minutes at room temperature in a 96-well plate. The reaction was stopped by adding 50 μL of stop solution and the plate was subsequently measured on a Mithras LB 940 microplate reader (Berthold Technologies) at 485 nm. Spontaneous and maximum LDH release controls were included in order to calculate the percentage of cytotoxicity. Macrophages were additionally stained with Trypan Blue (Sigma-Aldrich) as a second measure of cell viability.

### Phagocytosis Quantification Assay

To quantify phagocytic capacity, fluorescent polystyrene particles (Fluoresbrite YG carboxylate microspheres) were used as described by Leclerc *et al* [24]. In short, M2 macrophages were incubated with P-beads in a ratio of 10 beads to 1 cell for 90 minutes at 37°C. Subsequently cells were collected with a cell scraper and centrifuged at 1500 rpm for 10 minutes. Following centrifugation supernatant was discarded and cells were re-suspended in 100 μL culture medium or 100 μL culture medium and Trypan Blue (1:1) (Sigma-Aldrich). Internalisation of the beads was quantified by flow cytometry. Non-internalised beads emitted a red fluorescent signal after Trypan Blue quenching which was detected in the FL-3 channel where as internalised beads were detected in the FL-1 channel.

### Statistical Analysis

Differences were analysed using a Wilcoxon signed rank test (paired, non-parametric analysis) unless otherwise stated. Data was considered statistically significant at a p-value <0.05. Data are shown as cumulative results of levels obtained in all volunteers (means ± SEM).

## Results

### Hyperglycaemic culture conditions variably affect cytokine production from PBMCs

**Fig 1:**
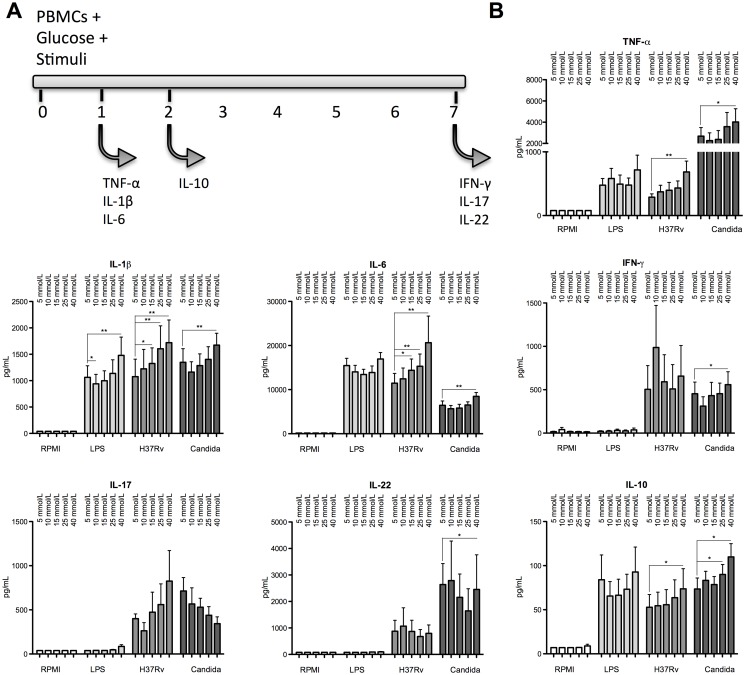
Cytokine production by PBMCs in response to antigenic stimuli in varying concentrations of glucose. (A) PBMCs were stimulated in the presence of 5 mmol/L to 40 mmol/L glucose with RPMI, H37Rv lysate (1 μg/mL), LPS (10 ng/mL) or heat-killed *Candida Albicans* (1 × 10^6^ microorganisms/mL). Cell culture supernatants were collected after 24 h, 48 h or 7 days. (B) Data are shown as mean ± SEM of supernatant TNF-α, IL-1β, IL-6, IFN-γ, IL-17, IL-22 and IL-10 levels obtained in ≥ 6 volunteers, *p<0.05, **p<0.01.

Little or no difference was seen in cytokine production following stimulation and culture of PBMCs in glucose concentrations ranging from 5 to 25 mmol/L, while culture in 40 mmol/L glucose mostly led to higher cytokine production (Fig. 1). Production of pro-inflammatory cytokines IL-6 and IL-1β significantly increased in a dose dependent manner upon H37Rv stimulation whereas TNF-α production only increased at the highest glucose concentrations of 40 mmol/L. Production of the anti-inflammatory cytokine IL-10 also increased upon H37Rv lysate and *Candida* stimulations in the presence of 25 mmol/L or 40 mmol/L glucose. In comparison, induction of T-cell cytokines showed a high degree of variability in response to varying glucose concentrations. Overall, compared to normal glucose concentrations of 5 mmol/L, 40 mmol/L glucose showed the most significant changes in cytokine production (all p-values <0.05).

### Macrophage differentiation in hyperglycaemic conditions leads to hyper-responsive cytokine production

**Fig 2:**
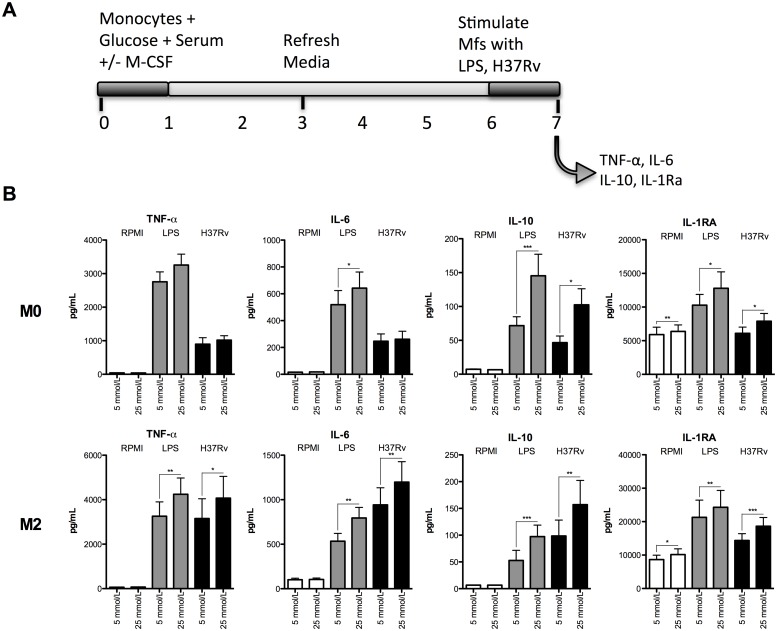
Cytokine production by M0 and M2 macrophages stimulated in varying concentrations of glucose. (A) Adherent monocytes were differentiated in either 5 or 25 mmol/L glucose into M0 macrophages (serum only) or M2 macrophages (M-CSF and serum) for 6 days and stimulated with RPMI, H37Rv lysate (1 μg/mL) or LPS (10 ng/mL). (B) Cell culture supernatants were collected and the pro-inflammatory cytokines TNF-α and IL-6 were measured along with the anti-inflammatory cytokines IL-10 and IL-1RA (n = 23). Data are shown as mean ± SEM, *p<0.05, **p<0.01 &, ***p<0.001.

The effect of hyperglycaemia on PBMC cytokine production was mild and mainly observed at the highest glucose concentrations (40 mmol/L). Both 25 mmol/L and 40 mmol/L of glucose can be observed in diabetes patients although the latter is rarely seen and is thus unlikely to account for the increase in TB susceptibility. We therefore proceeded by differentiating monocytes into M0 (serum derived) and M2 (M-CSF and serum derived) macrophages in the presence of 5 mmol/L glucose or the more clinically relevant hyperglycaemic condition of 25 mmol/L glucose. M2 macrophage differentiation was verified by assessing cell surface marker expression (CD14^+^/CD163^+^; S1 Fig.). Differentiated macrophages were stimulated with LPS or H37Rv lysate (Fig. 2A). Cytokine production was generally higher in macrophages differentiated under high glucose concentrations, although not all differences were statistically significant. Pro-inflammatory cytokines (TNF-α and IL-6) and anti-inflammatory cytokines (IL-10 and IL-1RA) from hyperglycaemic M2 macrophages were significantly increased after stimulation with H37Rv lysate and LPS. M0 macrophages displayed a similar pattern, except for TNF-α production. Sub-maximal concentrations of LPS and H37Rv were used to provide room for the potential boosting effects of hyperglycaemia on cytokine production.

Given the nature of these experiments the observed increase in cytokine production may have simply been a consequence of increased osmolarity during cell culture. To control for this, mannitol was used to achieve similar osmolarity in the 5 mmol/L and 25 mmol/L culture conditions (S2 Fig.). In most cases the elevated cytokine production from M0 and M2 hyperglycaemic macrophages, as seen in Fig. 2B, was reproducible under osmolarity controlled conditions. Thus the effects of hyperglycaemia on cytokine production cannot simply be explained by a difference in osmolarity.

### Hyperglycaemia does not affect the cytokine response to and the survival of *M*. *tuberculosis* in human macrophages

**Fig 3:**
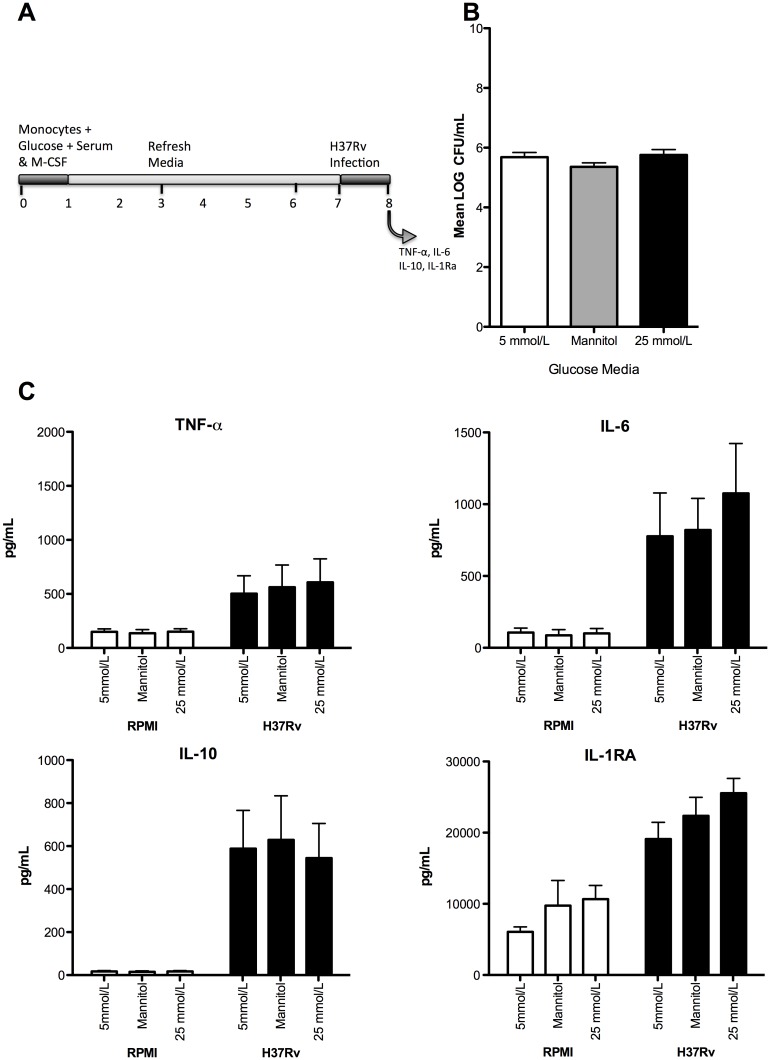
Effects of euglycaemic and hyperglycaemic culture conditions on H37RV infection and cytokine production in vitro. (A) CD14^+^ selected monocytes were differentiated into M2 macrophages in the presence of 5 mmol/L glucose, 5 mmol/L glucose and 20 mmol/L mannitol or 25 mmol/L glucose. The macrophages were infected for 1 hour with H37Rv at an MOI of 10. After infection the macrophages were washed and fresh media containing the different glucose media was added. After 24 hours supernatants were collected and the cells were lysed by osmotic pressure. (B) Cell lysates were serially diluted and plated on Middlebrook 7H10 agar. CFUs were counted after 2–3 weeks of growth at 37°C. Data are shown as mean ± SEM of two independent experiments (n = 4). (C) Pro-inflammatory cytokines TNF-α and IL-6 were measured along with the anti-inflammatory cytokines IL-10 and IL-1RA. Data are shown as mean ± SEM.

**Fig 4:**
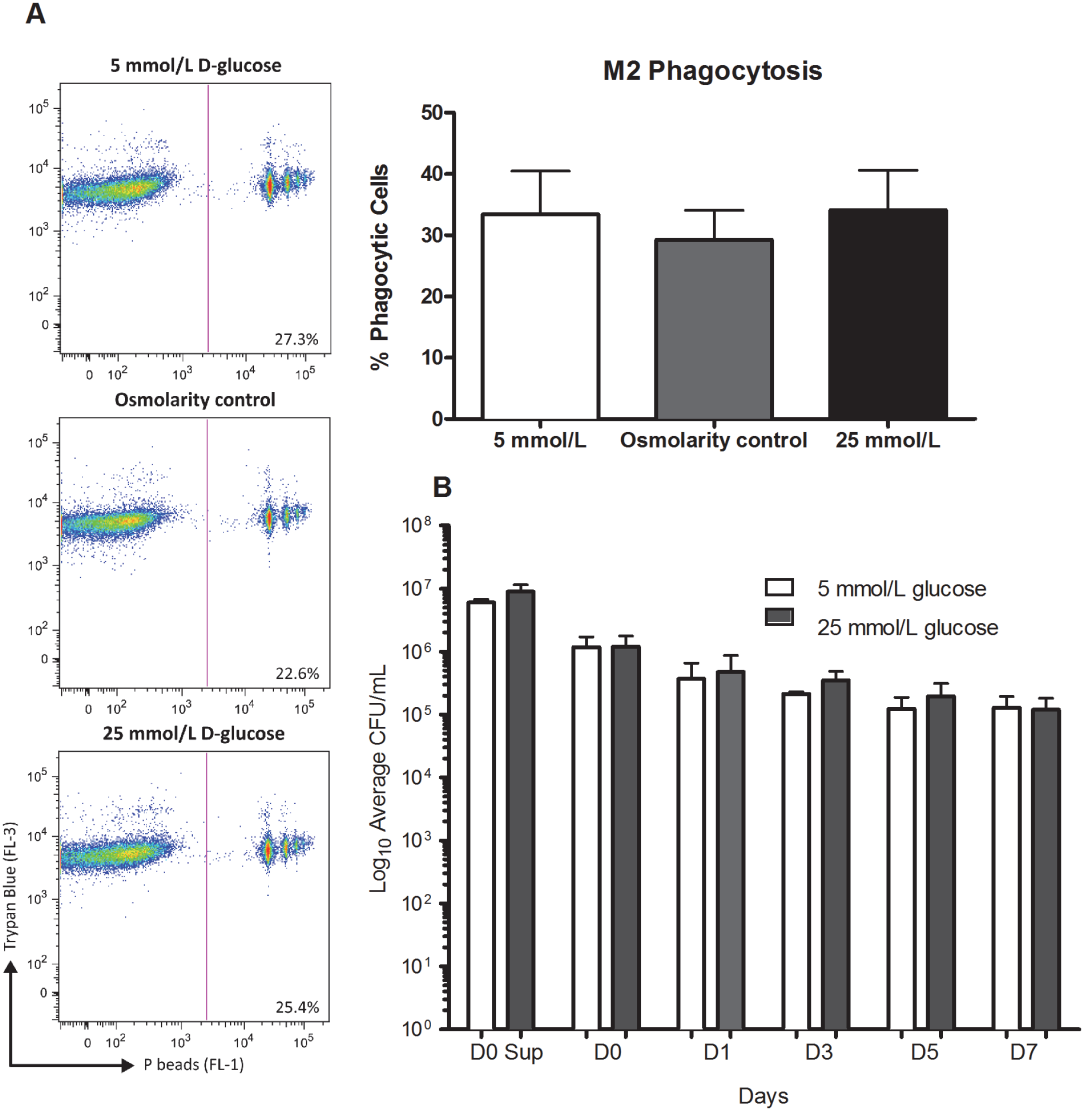
Effects of high glucose on phagocytic capacity of macrophages and infection with H37Rv. M2 macrophages differentiated in the presence of 5 or 25 mmol/L glucose were either incubated with P-beads and subjected to flow cytometry measurements to determine the percentage of phagocytic cells (A) or were infected for 1 hour with H37Rv at an MOI of 10:1 (B). After infection macrophages were washed three times and fresh RPMI containing the different glucose media was added. The first wash (Day 0 Sups) of each infection was collected and plated in serial dilutions to determine whether different amounts of bacilli were taken up by euglycaemic or hyperglycaemic macrophages. Simultaneously macrophages (D0) were lysed and plated for CFU counts. Infected macrophages were also lysed on Day 1 (D1), Day 3 (D3), Day 5 (D5) and Day 7 (D7) after infection. CFUs were counted at once after 2–3 weeks of growth at 37°C. Data are shown as mean ± SEM (n = 4).

After investigating the cytokine profiles of macrophages stimulated with either LPS or H37Rv lysate in the presence of varying glucose concentrations the effect of hyperglycaemia on the *in vitro* infection of M2 macrophages with the H37Rv strain of MTB was determined. M2 macrophages were used for the infection experiments as we have previously shown that this macrophage subtype is more adept in supporting mycobacterial survival and could therefore serve as the primary bacterial reservoir in the lungs during MTB infection [25]. After 24 h of infection no differences in CFU counts (Fig. 3B) or cytokine production (Fig. 3C) were observed between euglycaemic (5 mmol/L) and hyperglycaemic (25 mmol/L) macrophages. We also examined the effect of hyperglycaemia on the phagocytic capacity of macrophages and outgrowth of H37Rv after prolonged infection. M2 macrophages were able to control MTB growth as was demonstrated by a 1 log reduction in CFU over time (Fig. 4B). However, differentiation and stimulation of M2 macrophages in the presence of 5 or 25 mmol/L of glucose were not associated with differences in the phagocytosis of fluorescent P-beads (Fig. 4A), mycobacterial uptake (D0 Sups) and H37Rv survival throughout the course of infection (Fig. 4B). We assessed macrophage viability during infection by measuring LDH release and staining the cells with Trypan Blue and found no differences in viability between glucose conditions (S3 Fig.). Together these data demonstrate that hyperglycaemia influences neither *in vitro* H37Rv infection and survival nor the infection-induced cytokine response in human M2 macrophages.

## Discussion

Type 2 diabetes mellitus confers a three-fold increased risk for active tuberculosis, but the underlying immunological mechanisms have not been identified [1]. In this study we investigated the effects of hyperglycaemia on *in vitro* cytokine production and mycobacterial infection. Hyperglycaemia altered cytokine production by PBMCs and macrophages stimulated with H37Rv lysate, although significant effects were mainly observed at the higher end of the glucose concentration range. Alternatively, hyperglycaemia did not affect the phagocytic capacity of macrophages or their ability to control outgrowth of H37Rv over a period of time.

PBMCs incubated in high glucose concentrations produced higher levels of TNF-α, IL-1β and IL-6, whilst IFN-γ, IL-17A and IL-22 levels did not change. This suggests that hyperglycaemia mainly affects monocytes but not T cells. As a result, we investigated whether hyperglycaemia had a more specific effect on macrophage-derived cytokine production. An increase in IL-6, IL-10 and IL1RA levels was found when hyperglycaemic monocyte-derived macrophages were stimulated with MTB and LPS.

To our knowledge, no studies have presented data on the effects of high glucose levels on *in vitro* cytokine production in response to MTB. Previous studies that examined *ex vivo* cytokine production in diabetes patients with or without TB have provided conflicting results. Some studies have shown elevated production of pro-inflammatory cytokines from whole blood of patients with TB-DM whereas another study using whole blood and one using PBMCs reported defects in IFN-γ production in patients with TB-DM [19–21,26]. Interestingly, both the increase and decrease of pro-inflammatory cytokines were correlated to increased HbA1c levels [19,26]. Differences between studies can be explained by the use of different cell types and stimuli. In this study we chose to investigate M0 (serum derived) and M2 (M-CSF and serum derived) macrophages, as they most closely represent tissue resident macrophages such as alveolar macrophages, in which MTB dominantly resides. Furthermore, patient studies are often complicated by variations in age, HBA1c levels, metabolic perturbations, medication etc., making it difficult to specifically examine the effects of hyperglycaemia. For these reasons we chose to exclusively study the effects of hyperglycaemia *in vitro*.

In contrast to the effects on cytokine production, the capacity of macrophages to phagocytose P-beads remained unaltered under high glucose concentrations. In literature, several studies report findings that both support and contrast with our observations on the effects of hyperglycaemia or DM on the phagocytic capacity of macrophages. In a TB-DM animal model in particular no significant differences were found in the phagocytic capacity of alveolar macrophages from diabetic and non-diabetic rats subjected to aerosol infection with MTB [27,28]. In patients with DM the phagocytic function of macrophages and polymorphonuclear cells (PMN) is even more unclear [14,29–31]. In a recent study comparing patients with pulmonary TB, DM or the combination of TB and DM, no differences were found in the ability of PMNs to phagocytose, produce hydrogen peroxide or reduce nitroblue tetrazolium. In contrast, two studies from the same group using monocytes from patients with diabetes [32] showed a reduced association of MTB bacilli and reduced phagocytosis via the complement or Fc-γ receptor pathway, although this was not demonstrated in the context of MTB itself [33].

Similar to phagocytosis, no differences were found in MTB killing or outgrowth between hyperglycaemic and euglycaemic macrophages. To our knowledge no other data have been published on outgrowth of MTB in hyperglycaemic macrophages or macrophages from patients with DM. Of interest however is one study showing increased tuberculosis susceptibility in mice with streptozotocin-induced diabetes. In line with our study no differences in CFU counts were found in the lungs of acute diabetic mice. These results may indicate that hyperglycaemia may have long-term effects on susceptibility that are difficult to emulate *in vitro*. [27,34].

Several aspects and limitations of our studies should be considered when discussing the relatively mild effects of hyperglycaemia on the immune responses elicited by MTB. Firstly, although hyperglycaemia did not directly affect MTB survival in macrophages, it is possible that it does after longer periods of time, or contributes to increased susceptibility to infection indirectly through effects on other immune cells. Secondly, even though hyperglycaemia is regarded as a major hallmark of DM, the pathophysiology of the disease is not restricted to high glucose concentrations. Other physiological disturbances in DM such as hyperinsulinaemia, diabetic acidosis and metabolic changes have also been found to affect immune cell functions, [35–37] and studies to assess their effects on MTB-induced immune responses are needed. Thirdly, DM is often associated with diet-induced conditions like dyslipidaemia. As MTB has been found to modulate host lipogenic pathways to survive in macrophages [38] it is possible that changes in blood lipid levels or composition contribute to the increased risk of active TB disease in DM patients. Furthermore we cannot exclude that the unidentified donors used in these experiments suffered from co-morbidities such as diabetes, although the chance of that are <1.5% as described above. Finally, it is unclear how accurately our *in vitro* model of hyperglycaemia reflects the *in vivo* situation during DM.

In short, these *in vitro* studies in PBMC and macrophages suggest that hyperglycaemia cannot fully explain the increased susceptibility to MTB in DM patients. Further studies that explore a broader range of metabolic parameters and cell types are needed to unravel the precise mechanisms underlying the effect of DM on TB.

## Supporting Information

S1 Fig M2 macrophage surface marker expression. M2 macrophage differentiation was verified by analysing the cell surface expression of CD14 (FITC, clone HCD14) and CD163 (Alexa 647, clone RM3/1) by flow cytometry. The FACS plots display representative results for CD14^+^/CD163^+^ M2 macrophages (black) versus an unstained sample (grey).
(TIF)

S2 Fig Effects of osmolarity on cytokine production from differentiated macrophages. Monocytes were differentiated into M0 or M2 macrophages in the presence of 5 mmol/L glucose, 5 mmol/L glucose and 20 mmol/L mannitol, or 25 mmol/L glucose, and subsequently stimulated with RPMI, H37Rv lysate (1 μg/mL) or LPS (10 ng/mL). Cell culture supernatants were collected after 24 h and the pro-inflammatory cytokines TNF-α and IL-6 were measured along with the anti-inflammatory cytokines IL-10 and IL-1RA (n = 6). Data are shown as mean ± SEM, *p<0.05, **p<0.01 and ***p<0.001.
(TIFF)

S3 Fig M2 macrophage viability during prolonged H37Rv infection. M2 macrophages differentiated for 6 days in the presence of 5 or 25 mmol/L glucose were infected for 1 hour with H37Rv at an MOI of 10:1 After infection macrophages were washed three times and fresh RPMI containing the different glucose media was added. The percentage of cytotoxicity was assessed by measuring LDH release from day 0 to day 7 corrected using spontaneous and maximum LDH release controls per time point. Macrophages were additionally stained with Trypan Blue as a second measure of cell viability and the resulting percentage of viable cells is indicated in the graph with an * at each time point for both conditions (5 mmol/L: black; 25 mmol/L: red). Data are shown as mean ± SD (n = 2).
(TIF)
