# Supplementary figures and images for: The Effect of Hyperglycaemia on In Vitro Cytokine Production and Macrophage Infection with Mycobacterium tuberculosis
Source: PLoS One. 2015 Feb 9;10(2):e0117941. doi: 10.1371/journal.pone.0117941 (PMC4322041; doi:10.1371/journal.pone.0117941)

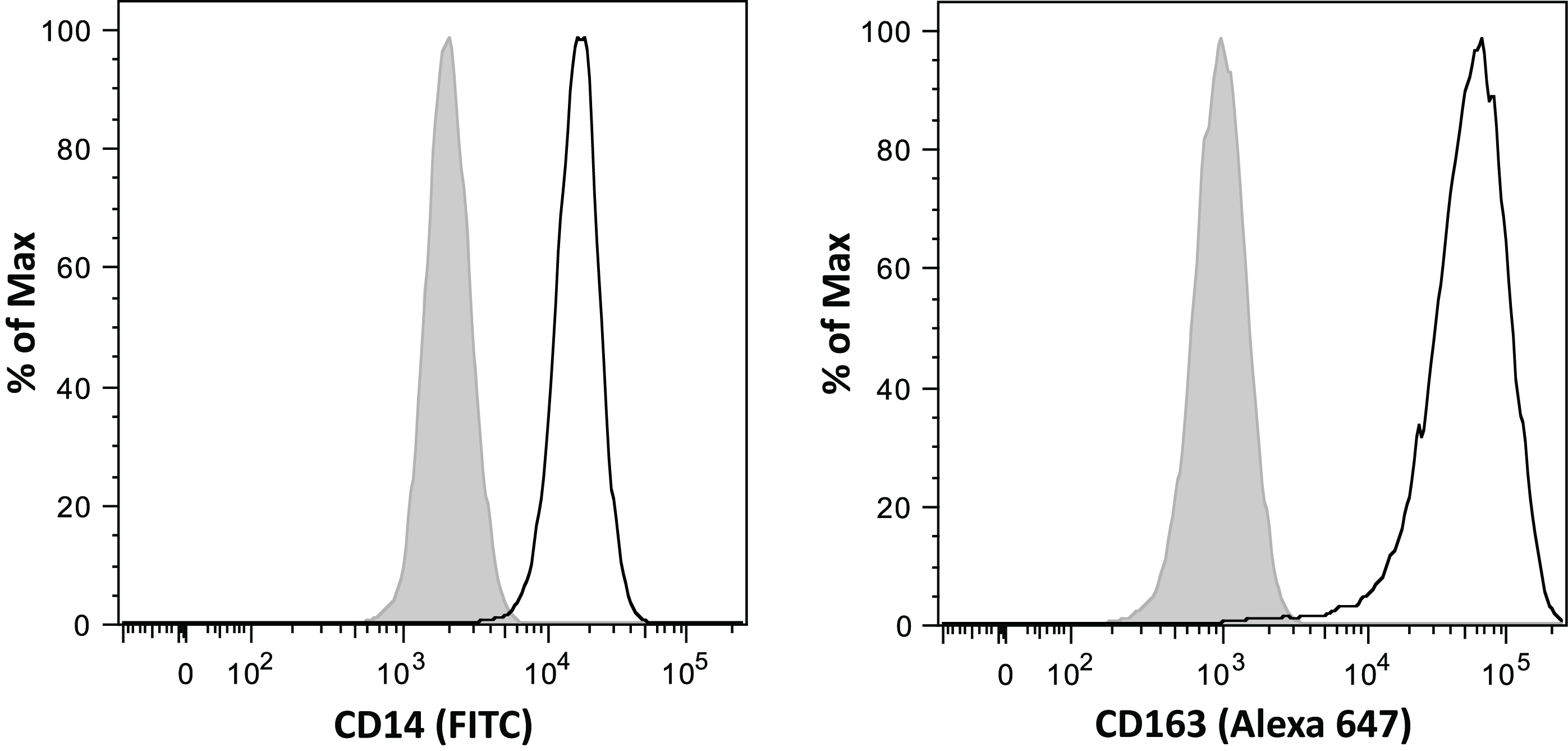

Supplement: S1 Fig — M2 macrophage differentiation was verified by analysing the cell surface expression of CD14 (FITC, clone HCD14) and CD163 (Alexa 647, clone RM3/1) by flow cytometry. The FACS plots display representative results for CD14+/CD163+ M2 macrophages (black) versus an unstained sample (grey). (TIF) [file pone.0117941.s001.tif]

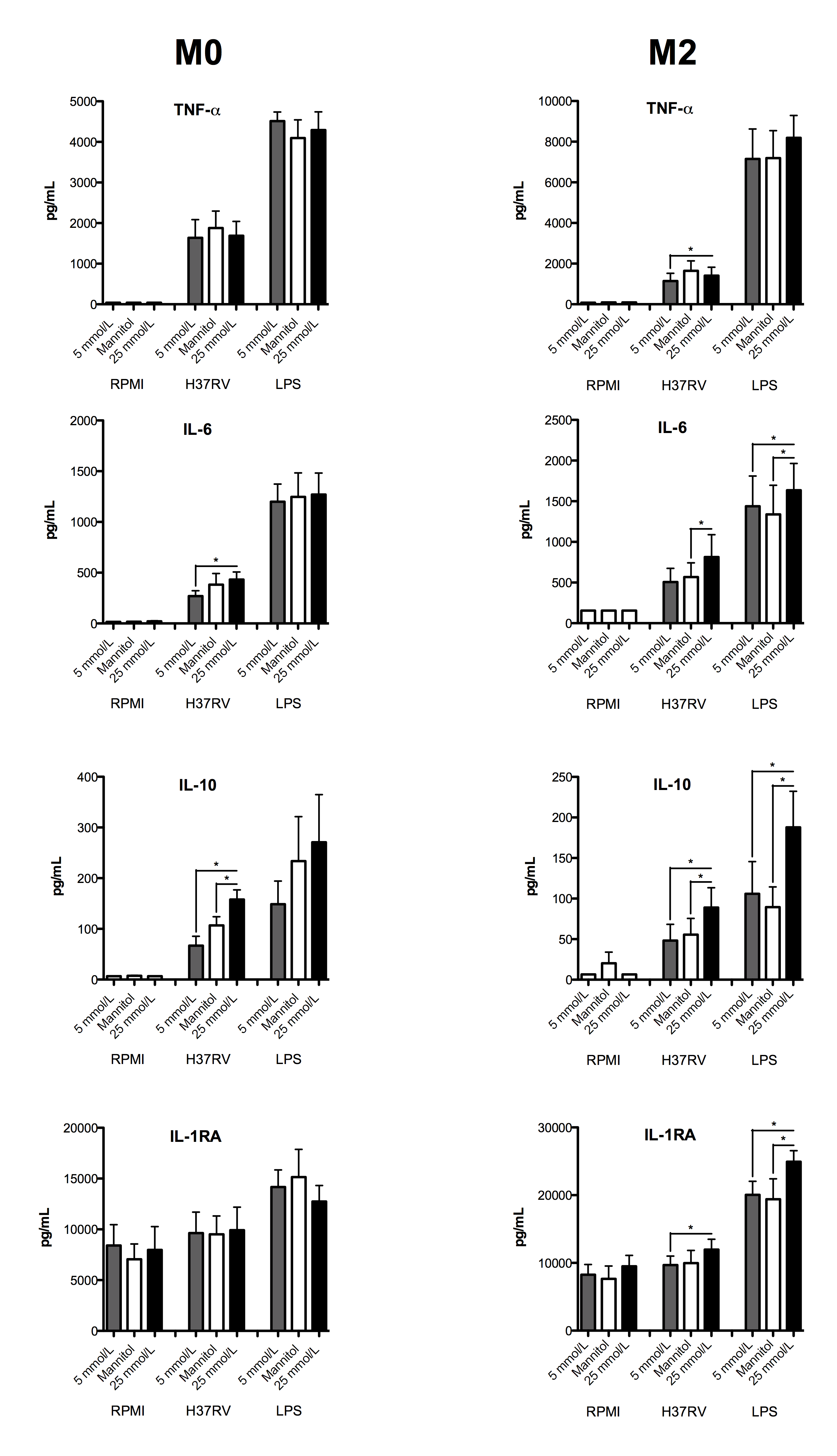

Supplement: S2 Fig — Monocytes were differentiated into M0 or M2 macrophages in the presence of 5 mmol/L glucose, 5 mmol/L glucose and 20 mmol/L mannitol, or 25 mmol/L glucose, and subsequently stimulated with RPMI, H37Rv lysate (1 μg/mL) or LPS (10 ng/mL). Cell culture supernatants were collected after 24 h and the pro-inflammatory cytokines TNF-α and IL-6 were measured along with the anti-inflammatory cytokines IL-10 and IL-1RA (n = 6). Data are shown as mean ± SEM, *p<0.05, **p<0.01 and ***p<0.001. (TIFF) [file pone.0117941.s002.tiff]
